# Colonic dialysis can influence gut flora to protect renal function in patients with pre-dialysis chronic kidney disease

**DOI:** 10.1038/s41598-021-91722-1

**Published:** 2021-06-17

**Authors:** Yueming Li, Minhui Dai, Jianqin Yan, Fang Liu, Xi Wang, Lizhen Lin, Mei Huang, Cuifang Li, Rui Wen, Jiao Qin, Hui Xu

**Affiliations:** 1 Department of Nephrology, Xiangya Hospital, Central South University, 87 Xiangya Road, Changsha, 410008 Hunan China; 2 Department of Nephrology, University of South China Affiliated Changsha Central Hospital, 161 Shaoshan Road, Changsha, 410004 Hunan China; 3 Department of Clinical Dietitian, Xiangya Hospital, Central South University, 87 Xiangya Road, Changsha, 410008 Hunan China; 4 Department of Anesthesiology, Xiangya Hospital, Central South University, 87 Xiangya Road, Changsha, 410008 Hunan China; 5 Health Management Center, Xiangya Hospital, Central South University, 87 Xiangya Road, Changsha, 410008 Hunan China; 6 Department of Endocrinology, Xiangya Hospital, Central South University, 87 Xiangya Road, Changsha, 410008 Hunan China

**Keywords:** Molecular biology, Nephrology

## Abstract

Chronic kidney disease (CKD) is a major public health burden around the world. The gut microbiome may contribute to CKD progression and serve as a promising therapeutic target. Colonic dialysis has long been used in China to help remove gut-derived toxins to delay CKD progression. Since disturbances in the gut biome may influence disease progression, we wondered whether colonic dialysis may mitigate the condition by influencing the biome. We compared the gut microbiota, based on 16S rRNA gene sequencing, in fecal samples of 25 patients with CKD (stages 3–5) who were receiving colonic dialysis(group CD), 25 outpatients with CKD not receiving colonic dialysis(group OP), and 34 healthy subjects(group HS). Richness of gut microbiota was similar between patients on colonic dialysis and healthy subjects, and richness in these two groups was significantly higher than that in patients not on colonic dialysis. Colonic dialysis also altered the profile of microbes in the gut of CKD patients, bringing it closer to the profile in healthy subjects. Colonic dialysis may protect renal function in pre-dialysis CKD by mitigating dysbiosis of gut microbiota.

## Introduction

Chronic kidney disease (CKD) involves chronic structural and functional disorder in the kidney, and it affects an estimated 10.8% of the Chinese population^1^. CKD progresses gradually from stage 1 to stage 5, with stages 1–2 involving the mildest impairment. To manage CKD in mild stages, patients consume a low-protein diet to reduce urinary protein excretion and they take medications to control blood glucose, blood pressure, blood lipids and uric acid^2^. Since clinical manifestations in early CKD stage may not be obvious, patients are often diagnosed when they are already in later stages^3^.

In China, colonic dialysis has long been applied to patients with pre-dialysis CKD (stages 3–5) as a simple, inexpensive procedure to remove uremic toxins. Even when patients are in later stages, the technique is often performed at the patient’s home by nephrologists. During colonic dialysis, osmotically balanced solution is injected into the colon to flush out gut-derived toxins and persistent stool. Several case studies and our own retrospective analysis suggest that colonic dialysis can improve symptoms of CKD^4–6^ and even delay progression of disease^7^. The technique may also improve renal function in patients with CKD in stages 3–5^7^, but how it does so is unclear.

CKD has been associated with dysbiosis of gut microbiota^8,9^, which has also been linked to other chronic diseases such as inflammatory bowel disease, colorectal cancer, diabetes, obesity and multiple system atrophy^10–14^. Such dysbiosis can also affect the pharmacokinetics of some drugs^15^. This dysbiosis may help explain several features of CKD, such as increased gastrointestinal secretion of urea, congestion and edema of the gut wall, slow bowel movement, metabolic acidosis. Dysbiosis may be exacerbated by CKD patients’ frequent use of antibiotics and lower dietary fiber consumption^16^. Moreover, gut microbiota dysbiosis impairs the gut barrier and generates toxins in the gut, which may exacerbate kidney disease^17^. This vicious circle is often called the “gut–kidney” axis, highlighting the gut microbiome as a potential cause of CKD progression and therefore a therapeutic target^18^.

We wondered whether colonic dialysis might mitigate CKD symptoms and delay its progression by altering the gut microbiome. Therefore we compared the gut microbiome between patients with CKD in stages 3–5 who were receiving dialysis or not, and we also compared these two patient groups with healthy controls.

## Methods

### Participants

This cross-sectional, prospective study was approved by the Medical Ethics Committee of the Xiangya Hospital of Central South University, Changsha, Hunan, China (approval 201806953). All subjects gave written informed consent for inclusion in the study, which was conducted in accordance with the Declaration of Helsinki.

Twenty-five patients with CKD in stages 3–5 receiving colonic dialysis(group CD) were matched according to age, sex and estimated glomerular filtration rate (eGFR) with 25 patients with CKD in stages 3–5 who were not receiving dialysis(group OP). The two groups of patients were matched in a 1:1 ratio. Patients were then matched to 34 healthy controls(group HS) according to age and sex in a 1:1.4 ratio. The three groups were recruited, respectively, from the Colonic Dialysis Unit, Outpatient Renal Clinic and Physical Examination Center of Xiangya Hospital of Central South University between October 2018 and June 2019.

The patients in the CD group had to satisfy the following inclusion criteria: (1) CKD was diagnosed by a nephrologist at our hospital based on the “Kidney Disease: Improving Global Outcomes” standards^19^; (2) patients were determined to have stage 3–5 disease, based on the eGFR formula (CKD-EPI)^20^; (3) patients had not undergone peritoneal dialysis, hemodialysis, or kidney transplantation; and (4) patients had already been receiving colonic dialysis for more than 1 month. The patients in the OP group who were not on colonic dialysis had to satisfy criteria (1)–(3). Healthy subjects(HS) had to have no history of kidney injury.

Subjects were excluded from the study if they were pregnant or lactating; if they had taken immunosuppressants, antibiotics, prebiotics, probiotics or adsorbents of intestinal toxins within 4 weeks before the study; or if they had tumors or inflammatory bowel disease.

### Data and sample collection

Each participant provided a fresh stool sample in a sterile 5-ml tube (LangFu Biological, Shanghai, China), which was transported to the laboratory within 10 min and stored at − 80 °C until fecal DNA extraction and sequencing (see below). Clinical information including baseline characteristics, laboratory values and medical history were obtained from medical records.

### Colonic dialysis

Colonic dialysate was prepared from concentrated Dialysate A (catalog no. 6ATA06, prepared by Xiangya Hospital, Central South University), dialysate B (catalog no. 6ATB02, prepared by Xiangya Hospital) and reverse osmosis water (prepared by Xiangya Hospital) in a ratio of 1:1.225:32.775. The dialysate temperature was warmed to 34–38 °C and delivered using a Zhili Colonic Therapy System (Model BT600-02, Sunny Medical, Beijing, China). Patients were asked to empty their bladder before dialysis in order to reduce discomfort. During dialysis, patients were asked to remain in a left recumbent position with their two knees bent. A single-use double lumen rectal catheter (Huaxia Medical Equipment, Jiangsu China) was inserted through the anus into the colon to an intubation depth of 65–75 cm, reaching the ascending colon. Colonic dialysate was irrigated into the colon through the inner cavity for 10 s. After allowing the dialysate to remain in the patient for 8–10 min, the solution and wastes were drained out of the colon through the external cavity for 18–20 s. Both cavities had sided-holes to prevent blockage. During the procedure, the dialysate was changed repeatedly until the end of dialysis, and the pressure in the lumen was 50–65 kPA during irrigation and 3–8 kPA during drainage. Each colonic dialysis session usually lasted 1 h, and the total volume of dialysate was 15–16 L.

### Fecal DNA extraction and 16S rRNA sequencing

Microbial DNA was extracted using the QIAamp® DNA Stool Mini Kit (Qiagen, Hilden, Germany). The V3-V4 hypervariable region of the 16S rRNA gene was amplified from genomic DNA using primers 341F (CCTACGGGNGGCWGCAG) and 805R (GACTACHVGGGTATCTAATCC). The amplification products were purified using Agencourt AMPure XP Beads (Beckman Coulter Genomics, MA, USA) according to the manufacturer’s instructions and quantified using the Qubit quantification system (Thermo Scientific, Wilmington, DE, US). Amplicons were sequenced on the MiSeq system (Illumina, CA, USA) using the Version 2 Reagent Kit (Illumina). Automated cluster generation and 2 × 250 bp paired-end sequencing with dual-index reads were performed.

Fastq files were de-multiplexed using MiSeq Controller Software (Illumina). Sequences were trimmed to remove amplification primers, diversity spacers, and sequencing adapters, then the resulting sequences were merge-paired and quality-filtered using USEARCH. UPARSE was used to cluster operational taxonomic units (OTUs) . Taxonomy of the OTUs was assigned and sequences were aligned using an RDP classifier. Phylogeny and other aspects of OTUs were analyzed using QIIME version 1.9.0(http://qiime.org/). Each OTU sequence was compared to the Greenenes (13-5) database, and microbiota were classified to the level of phylum, class, order, family, genus and/or species. The most abundant four species in each phylum were used to generate column accumulation diagrams showing the relative abundance of different species. The 50 top OTUs across all species were clustered and used to generate a heat map.

### Statistical analysis

Differences in clinicodemographic data across the three groups were assessed for significance in SPSS 22.0 (IBM, Chicago, IL, USA) using one-way ANOVA if the data showed a normal distribution and homogeneous variance; otherwise, a non-parametric test was used. We used the Shannon and Simpson indices to compare the diversity of gut flora among the three groups. Linear discriminant analysis effect size (LEfSe) was calculated using the Galaxy web-based interface (http://huttenhower.sph.harvard.edu/galaxy) in order to identify bacterial biomarkers of the three groups. The effect size cutoff was set to 2.0. Gut flora genera differing across the groups were identified based on the T test because of the small sample. Differences associated with *p* < 0.05 were considered significant.

### Ethical approval

This cross-sectional, prospective study was approved by the Medical Ethics Committee of the Xiangya Hospital of Central South University, Changsha, Hunan, China (approval 201806953). All subjects gave written informed consent for inclusion in the study, which was conducted in accordance with the Declaration of Helsinki.

## Results

### Participant characteristics

The study involved 34 healthy subjects (16 men, mean age 52.62 ± 6.68 year), 25 CKD patients on colonic dialysis (12 men, mean age 56.24 ± 11.7 year), and 25 CKD patients not on colonic dialysis (13 men, mean age 51.08 ± 8.58 year). Both groups of patients showed significantly higher systolic blood pressure and levels of urea, uric acid and triglycerides than healthy subjects, but significantly lower levels of hemoglobin and albumin (Table 1). The two patient groups were similar in medication history.

**Table 1:** Baseline characteristics of study participants.

Characteristic	Healthy subjects	Patients with chronic kidney disease	
		Colonic dialysis	No dialysis
N	34	25	25
Age (year)	52.62 ± 6.68	56.24 ± 11.7	51.08 ± 8.58
Male	16 (47.1)	12 (48)	13(52)
SBP (mmHg)	119.63 ± 18.69	138.72 ± 17.37*	136.04 ± 17.92#
DBP (mmHg)	77.50 ± 12.00	81.96 ± 9.01	79.32 ± 10.08
**CKD stage**			
3–4 (eGFR 15–59)	0	10	13
5 (eGFR < 15)	0	15	12
**Primary disease**			
CGN	0	9	16
DM	0	5	6
HT	0	7	0
SLE	0	1	0
AP	0	1	0
DPKD	0	0	1
Other	0	2	2
Colonic dialysis duration (year)	–	3.26 ± 2.52(0.6–8.5)	–
Colonic dialysis frequency (times per week)	–	3.33 ± 1.39(1–7)	–
**Laboratory parameters**			
Hb (g/L)	141.97 ± 14.35	107.75 ± 20.87*	96.92 ± 25.50^#^
ALB(g/L)	46.06 ± 2.50	39.67 ± 5.59*	38.94 ± 8.00^#^
LDL (mmol/L)	3.29 ± 0.69	3.00 ± 0.76	3.16 ± 0.77
TG (mmol/L)	1.57 ± 0.90	2.30 ± 1.38*	2.19 ± 1.45^#^
TC (mmol/L)	5.27 ± 0.90	4.74 ± 1.18	5.06 ± 1.03
Glu (mmol/L)	5.98 ± 1.86	5.46 ± 1.29	6.36 ± 3.61
Urea (mmol/L)	5.29 ± 1.32	17.11 ± 6.93	16.37 ± 6.74
UA (mmol/L)	334.01 ± 80.22	431.18 ± 104.95*	443.14 ± 103.85^#^
K (mmol/L)	–	4.52 ± 0.60	4.64 ± 0.71
Ca (mmol/L)	–	2.21 ± 0.15	2.20 ± 0.19
P (mmol/L)	–	1.41 ± 0.30	1.46 ± 0.50
Mg (mmol/L)	–	0.86 ± 0.15	0.84 ± 0.10
**Qualitative determination of urinary protein**			
Negative	30	1	1
Microscale	3	3	0
Positive	1	21	24
**Medications at baseline**			
Calcium channel-blockers	–	17	11
ACE inhibitors/ARBs	–	8	9
Beta-blockers	–	10	7
Diuretics	–	2	2
Iron agents	–	3	5
Phosphate binders	–	0	0
EPO	–	5	7
Anticoagulants	–	7	6
Lipid-lowering drugs	–	6	5
Antidiabetic drugs	–	4	4
Other BP-lowering drugs	–	6	1

Values are n, n (%), or mean ± SD, unless otherwise noted.

*SBP* systolic blood pressure, *DPB* diastolic blood pressure, *CKD* chronic kidney disease, *CGN* chronic glomerulonephritis, *DM* diabetic mellitus, *HT* hypertension, *SLE* systemic lupus erythematosus, *AP* allergic purpura, *PKD* polycystic kidney disease, *Hb* hemoglobin, *ALB* albumin, *LDL* low density lipop-rotein, *TG* triglyceride, *TC* cholesterol, *Glu* glucose, *UA* uric acid, *K* potassium, *Ca* calcium, *P* phosphorus, *Mg* magnesium, *EPO* Erythropoietin. *p < 0.05 versus healthy subjects, ^#^*p* < 0.05 versus healthy subjects.

A total of 84 stool samples were collected from the 84 study subjects and analyzed by high-throughput 16S rRNA sequencing.

### Gut microbiome profiling

**Figure 1:**
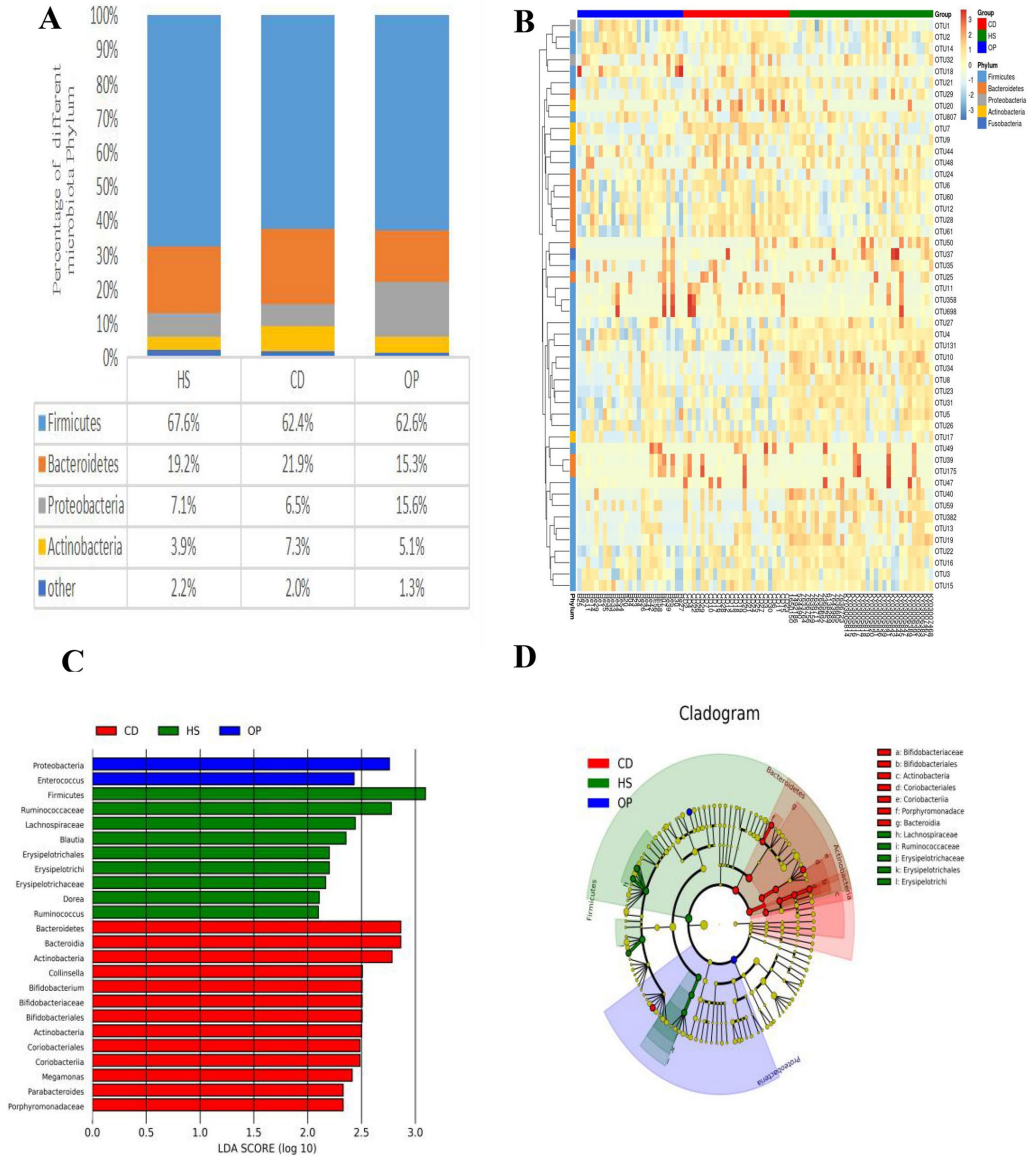
Differences in gut microbiome biodiversity across the two patient groups and one group of healthy controls. The three populations were CKD patients on colonic dialysis (CD, red) or not (OP, blue), as well as healthy controls (HS, green). (**A**) Gut microbiome profiles at phylum level. (**B**) The top 50 operational taxonomic units (OTUs) of all samples were used to generate a heat map. The x-axis represents the sample; the y-axis, OTUs. Color shading reflects OTU abundance. (**C**) Analysis of linear discriminant analysis effect size (LEfSe) was performed on the three groups. The x-axis indicates the linear discriminant analysis (LDA) score; the y-axis, the taxa that help distinguish the three groups from one another. The larger the value is, the greater is the difference. Different colors represent different groups. (**D**) Cladogram, in which the small circles radiating from the inside to the outside represent the classification level of the species at the level of phylum, class, order, family, and genus. The diameter of the small circle represents relative abundance. The nodes of different colors in the phylogenetic tree are the microbial groups that distinguish the given group from the two others. The closeness and partial overlap of green and red areas in the phylogenetic tree suggest that the intestinal microbiome of CKD patients on colonic dialysis was more similar to that of healthy subjects than to that of patients not on dialysis.

We compared the relative taxon abundances for the phyla Firmicutes, Bacteroidetes, Proteobacteria, and Actinobacteria across all three groups. Firmicutes dominated the gut microbiome at the phylum level in all three groups. Patients not on dialysis showed a significantly higher proportion of Proteobacteria and significantly lower proportion of Bacteroidetes than the patients on dialysis or healthy subjects (Fig. 1A). A heat map showing the abundance of the top 50 OTUs indicated that the gut microbiota of patients not on dialysis differed from that of healthy controls (Fig. 1B). LEfSe analysis confirmed that the abundances of Proteobacteria and Enterococcus were significantly different from those in the other two groups (Fig. 1C,D). The abundances of Firmicutes and Ruminocaccae differed significantly between healthy subjects and the two patient groups, while the abundances of Bacteroidetes, Bacteroidia and Actinobacteria differed significantly between patients on dialysis and the other two groups. Altogether, the gut microbiome of patients on dialysis was more similar to that of healthy subjects than to that of patients not on dialysis.

### Influence of colonic dialysis on richness of the gut microbiome in CKD patients

**Figure 2:**
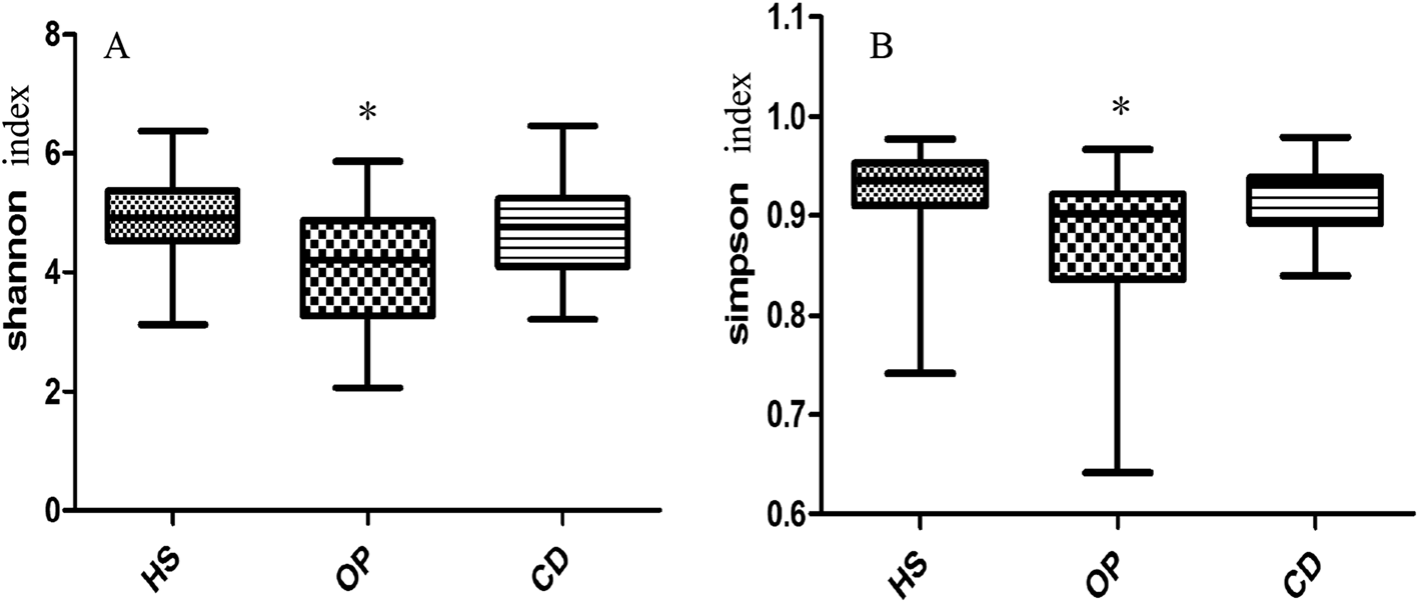
Richness of the gut microbiome in the three groups.The three populations were CKD patients on colonic dialysis (CD) or not (OP), as well as healthy controls (HS). (**A**) Shannon index of the gut microbiome. (**B**) Simpson index of the gut microbiome. *p < 0.05 in comparison to HS group or CD group.

Colonic dialysis was associated with greater richness of microbiota, similar to the richness in healthy individuals (Fig. 2). Colonic dialysis was associated with lower abundance of bacteria that possess uricase (e.g. Citrobacter of Enterobacteriaceae, Rothia of Micrococcaceae) and urease, or that produce indoxyl sulfate or p-cresyl sulfate (Table 2); it was also associated with higher abundance of bacteria that produce short-chain fatty acids (e.g. Dorea, Dialister, Phascolarctobacterium, Lachnospira). Colonic dialysis was associated with lower abundances of aerobes and facultative anaerobes, including Enterococcus, Granulicatella, Neisseria, Citrobacter and Rothia; but higher abundances of some anaerobes, including Anaerotruncus, Dorea, Dialister and Sutteralla.

**Table 2:** Taxa in the gut microbiome differing significantly between CKD patients not on colonic dialysis and healthy controls.

Phylum	Family	Genus	Change in abundance, relative to controls
Firmicutes	Enterococcaceae	Enterococcus	↑
		Other	↑
	Ruminococcaceae	Anaerotruncus	**↓**
		Other	**↓**
	Lachnospiraceae	Dorea	**↓**
		Other	**↓**
		Lachnospira	**↓**
	Aerococcaceae	Other	↑
	Carnobacteriaceae	Granulicatella	↑
	Veillonellaceae	Dialister	↓
		Phascolarctobacterium	↓
Proteobacteria	Alcaligenaceae	Sutteralla	↓
	Neisseriaceae	Neisseria	↑
	Phyllobacteriaceae	Other	↑
	Enterobacteriaceae	Citrobacter	↑
Actinobacteria	Micrococcaceae	Rothia	↑

Colonic dialysis was associated with higher abundance of microbiota that produce short-chain fatty acids or that are anaerobes, including Dialister, Phascolarctobacterium, Bacteroides, Collinsella and Bifidobacterium (Table 3). It was associated with lower abundance of microbes that possess urease and that produce indoxyl sulfate or p-cresyl sulfate, including unclassified genera of Enterobacteriaceae.

**Table 3:** Taxa in the gut microbiome differing significantly between CKD patients receiving colonic dialysis or not.

Phylum	Family	Genus	Change in abundance, relative to patients not on colonic dialysis
Firmicutes	Veillonellaceae	Dialister	↑
		Phascolarctobacterium	↑
	Enterococcaceae	Enterococcus	↓
	Carnobacteriaceae	Granulicatella	↓
	Gemellaceae	Other	↓
	Erysipelotrichaceae	Bulleidia	↓
Proteobacteria	Alcaligenaceae	Sutteralla	↑
		Achromobacter	↓
	Enterobacteriaceae	Other	↓
	Desulfovibrionaceae	Bilophila	↑
	Rhizobiales (Order)	Other	↓
	Other (Family)		
Actinobacteria	Coriobateriaceae	Collinsella	↑
	Bifidobacteriaceae	Bifidobacterium	↑
Bacteroidetes	Porphyromonadaceae	Parabacteroides	↑
	Bacteroidaceae	Bacteroides	↑

**Figure 3:**
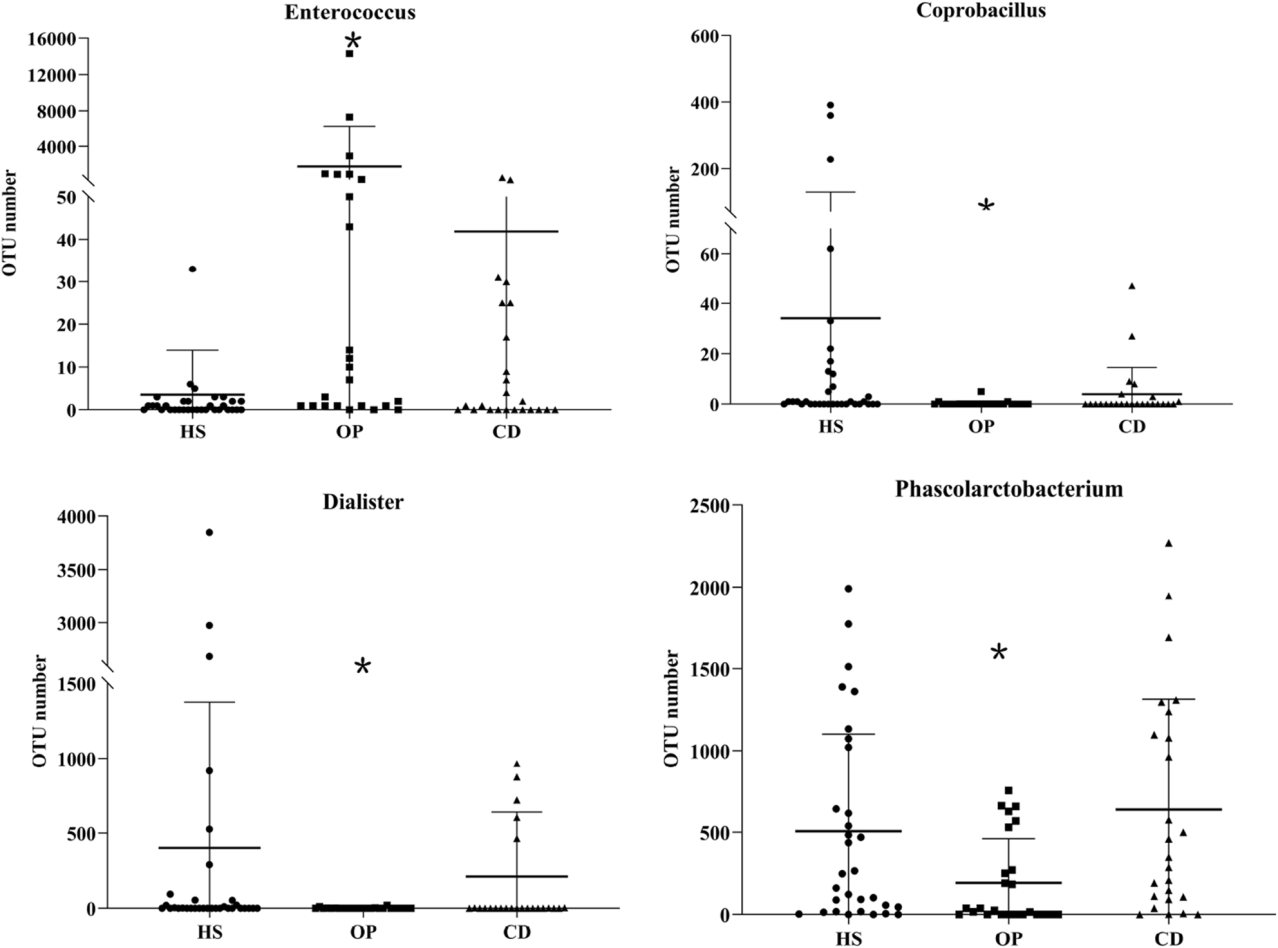
Effects of colonic dialysis on CKD-induced imbalance of the gut microbiome. *OTU* operational taxonomic unit.

Colonic dialysis was associated with significantly lower abundance of Enterococcus and Coprobacillus, and significantly higher abundance of Dialister and Phascolarctobacterium (Fig. 3).

## Discussion

### Summary

In this study, we investigated differences in gut microbiota between patients with CKD in stage 3–5 who were on colonic dialysis or not, as well as differences between both groups of patients and healthy subjects. Our results indicate that CKD is associated with lower richness of gut microbiota, consistent with previous work^21^. Greater diversity of gut microbiota may be associated with better health^22^. We provide here the first evidence that colonic dialysis may increase the diversity of the gut microbiome in patients with pre-dialysis CKD, even up to the levels observed in healthy individuals. Thus, colonic dialysis may mitigate CKD-associated dysbiosis of the gut microbiome. It may exert these effects by promoting defecation, reducing toxin accumulation due to slow transit time through the colon, and making the colon more hospitable for healthy intestinal flora.

### Comparison with existing literature

We found that CKD patients not receiving colonic dialysis had significantly higher abundances of Proteobacteria as well as aerobic and facultative anaerobic bacteria than healthy subjects, consistent with previous work^23–25^. An increase in the proportion of Proteobacteria is considered a sign of imbalance in gut flora and may be associated with inflammation^26^. Indeed, aerobic or facultative anaerobic bacteria may be less desirable inhabitants of the gut than certain anaerobic bacteria^27^.

We also found that CKD patients not receiving colonic dialysis had significantly higher abundances of Micrococcacea, Enterobacteriaceae Aerococcaceae and Enterococcus than healthy subjects, consistent with previous studies^9,28^. These bacteria contain enzymes related to the production of uremic toxins, such as urea, Indole sulfate, p-Cresol sulfate and phenyl sulfate, which can promote inflammation and CKD progression^29–35^.

Our study provides the first evidence that CKD is associated with significant decreases in the abundances of Anaerotruncus and Phascolarctobacterium, which produce short-chain fatty acids. These fatty acids are nutrients for intestinal epithelial cells, so their decline may cause intestinal epithelial dysfunction, reduce the number of intestinal epithelial cells, promote the passage of potentially harmful intestinal contents or metabolites into the circulatory system, trigger systemic inflammation and aggravate CKD^36^. Short-chain fatty acids also regulate blood pressure by activating G protein-coupled receptors such as GPR41 and GPR4^37^, and they may exert anti-inflammatory effects that protect kidneys from injury^38^. We found that colonic dialysis increased the richness of the anaerobe Coprobacillus in CKD patients, and this bacterium produces short-chain fatty acids from glucose^39^. It is tempting to speculate that Coprobacillus may slow CKD progression by regulating blood pressure and/or by exerting anti-inflammatory effects that protect the kidneys. Further studies should explore these possibilities.

Some of the bacteria whose abundances were elevated in our patients have not previously been reported as elevated in CKD, such as Citrobacter and Phyllobactriaceae. These differences from previous studies may reflect that our patients were Chinese, whereas previous studies examined other ethnic groups^25^. Another potential explanation is that patients in other studies had undergone hemodialysis or peritoneal dialysis^40^, while our patients had not. Another explanation is that much of the current data on gut microbiome and CKD have come from animal studies^33,41^, which may not always accurately reflect the situation in humans.

Our results suggest that colonic dialysis may be able to reverse the CKD-associated decrease in abundance of Parabacteroides, Bifidobacteria, Phascolarctobacteria and Dialister. Bifidobacteria are recognized probiotics, and increases in the abundance of Bifidobacteria and Lactobacilli in feces correlate with improvement in inflammatory indices, iron status and stabilization of iPTH^35^. Conversely, colonic dialysis appears to reduce the abundance of Enterococcus and an unclassified genus in Enterobacteriaceae. Whether these reductions help explain the therapeutic effects of dialysis in CKD should be explored.

Our CKD patients not on dialysis showed significantly lower abundance of Parabacteroides than healthy subjects, which is the opposite of what another study reported^42^. This difference might be related to diet, geographic region, disease stage or other factors.

### Strengths and limitations

Our study provides evidence that colonic dialysis can mitigate the symptoms of CKD in stages 3–5 by partially restoring healthy gut microbiota. Our results are likely to be reliable because we matched patients and healthy subjects on variables that can affect gut flora, including age, sex and use of gut-detoxifying drugs. Nevertheless, our results should be interpreted with caution given several limitations of the study. First, the patients’ diet was not taken into account, so we cannot exclude dietary effects on gut flora composition. Second, we did not assay levels of gut microbiome metabolites in plasma, so we had to rely on the literature to predict whether observed changes in abundance of certain bacteria is likely to be helpful or harmful. Third, studies are needed to examine whether colonic dialysis causes excessive discomfort to patients, or whether it damages the intestinal mucosa enough to cause malnutrition or inflammatory reactions, which can be the case with peritoneal dialysis^43^. Fourth, our sample came from a single medical center, and intestinal flora can differ across individuals of the same ethnic group living in different geographic areas^44^. Our results should be verified and extended in studies spanning different ethnicities and geographic regions.

## Conclusion

Our results suggest that colonic dialysis may preserve renal function in pre-dialysis CKD by mitigating dysbiosis of gut microbiota. As an effective treatment to improve gut flora imbalance, colonic dialysis may be useful against other diseases related to dysbiosis of gut microbiota. Future studies should also explore whether there are differences in the level of gut microbiome-related metabolites and the state of inflammation between CKD patients on colonic dialysis and CKD patients not on colonic dialysis.
